# The complete mitochondrial genome of *Luehdorfia chinensis* Leech (Lepidoptera: Papilionidae) from China

**DOI:** 10.1080/23802359.2016.1155084

**Published:** 2016-03-28

**Authors:** Yan Dong, Li-Xin Zhu, Chang-Bao Wang, Min Zhang, Pei-Pei Ding

**Affiliations:** College of Biology and Food Engineering, Chuzhou University, Chuzhou, China

**Keywords:** Chinese Luehdorfia Butterfly, *Luehdorfia chinensis*, mitogenome, phylogenetic analyses

## Abstract

The complete mitochondrial genome of *Luehdorfia chinensis* that is endemic to China is determined. The circular genome is 15 550 bp in length and comprises 13 protein-coding genes (PCGs), 22 tRNAs, two rRNA gene and a control region. Gene order is identical to that of the putative ancestral arrangement of insects. The nucleotide composition of heavy strand is A (40.3%), C (11.2%), T (41.1%) and G (7.3%). All PCGs start with a typical ATN codon except for the gene COI that uses AAC as the start codon. Bayesian analyses support the monophyly of genus *Papilio* and the sister relationship of *Luehdorfia chinensis* and *L. taibai*.

Because of environmental change and human hunt, some of the swallowtail butterflies are regarded as the most severely threatened species group in China. The species of *Luehdorfia chinensis* is an endemic butterfly to China, distributed along the middle and lower reaches of the Yangtze River and in Qinling Mountains in Central China (Yuan et al. 1998). It is a poorly known species, its population status meets the following items of the International Union for Conservation of Nature (IUCN) new criteria for endangered species: INSUFFICIENTLY KNOWN (Collins & Morris 1985; Groombridge 1993) and the species is classified as wildlife under second class protection by Chinese government (Yuan et al. 1998). There are some studies for taxonomy, morphology and biology about *L. chinensis* while complete mitochondrial genome of it is not recovered (Wang et al. 2010). Liu et al. (2013) reported the uncomplete mitochondrial genome of *L. chinensis* lacking three protein-coding genes (PCGs), two rRNA genes, AT-rich region and some tRNAs.

The mitochondrial genome of *L. chinensis* specimens was obtained from Langya Mountain of Chuzhou, Anhui Province, China (32°16′N, 118°16′E). Total genomic DNA was extracted from the two legs using the DNeasy tissue kit (Qiagen China, Shanghai). The entire mitogenome was amplified in three short and seven long overlapping fragments in the present study. Takara *Taq* and Takara LA *Taq* (Takara Biomedical, Japan) were used for polymerase chain reaction (PCR) amplification and the purified PCR products were sequenced directly with the amplified primers and internal walking primers in both directions.

The mitogenome of Chinese Luehdorfia Butterfly is a circular molecule, 15 550 bp in size, under GenBank accession number KU360130. The complete mitogenome of *L. chinensis* consisted of two rRNAs, 22 tRNAs, 13 PCGs and one major noncoding region, referred to as the A + T-rich region in insects. The starting codons inferred for the 13 PCGs are the conventional starting types ATN, except that the *cox1* and *nad5*. The base compositions of *L. chinensis* mtDNA had a high AT tendency. Nucleotide compositions of A (40.3%), T (41.1%), G (7.3%) and C (11.2%) are highly A-T biased (81.4%), which corresponds well to the A-T bias generally observed in other insect mitogenomes, ranging from 69.5% to 84.9% (Crozier & Crozier 1993; Dotson & Beard 2001). The higher A + T content of *L. chinensis* mtDNA was observed in the control region (96.8%). Gene order is identical to that of the putative ancestral arrangement of insects (Boore 1999). *L. chinensis* mitogenome also included intergenic spacers and overlapping regions in common with other lepidopteran species.

Mitogenomic phylogenies have revealed well-supported relationships for many eukaryote groups. Based on nucleotide sequences of 13 PCGs, the following clades were highly supported in maximum likelihood analyses (Figure 1): 1. *Luehdorfia chinensis *+* L. taibai*; 2. *Papilio Maraho* + *P. machaon *+* P. xuthus* +* P. bianor *+* P. maackii* +* P. Syfanius*.

**Figure 1.**
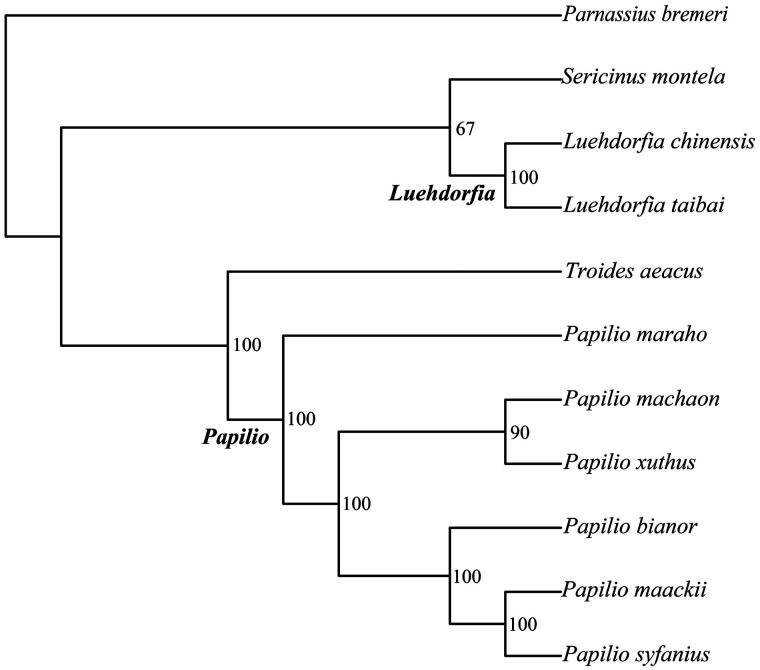
Phylogenetic relationships of 11 butterflies inferred from the sequences of 13 protein-coding genes. The values at each node are ML bootstrap values. Mitogenome of 11 butterflies were obtained from GenBank, including *Luehdorfia chinensis* (KU360130), *Luehdorfia taibai* (KC952673), *Sericinus montela* (HQ259122), *Troides aeacus* (EU625344), *Papilio maraho* (NC_014055), *Papilio machaon* (HM243594), *Papilio xuthus* (EF621724), *Papilio bianor* (KC433409), *Papilio maackii* (KC433408), *Papilio syfanius* (KJ396621) and *Parnassius bremeri* (NC_014053).
